# On the Treatment of Poisoned Wounds, Etc.

**Published:** 1854-08

**Authors:** Daniel Brainard

**Affiliations:** Prof. of Surgery in the Medical College at Chicago, Corresponding Member of the Societe de Chirurgic de Paris, etc., etc.


THE NORTH-WESTERN

MEDICAL AND SURGICAL JOURNAL.

NEW SERIES.

VOL. III. AUGUST, 1854. NO. 8.

### ORIGINAL COMMUNICATIONS.

ARTICLE I.—*On the Treatment of Poisoned Wounds, by the Application of Cupping Glasses and the Sub-Cutaneous Infiltration of Solutions of Iodine*—By DANIEL BRAINARD, M. D., Prof. of Surgery in the Medical College at Chicago, Corresponding Member of the Societe de Chirurgie de Paris, etc., etc (Being the Annual Address before the State Medical Society of Illinois, delivered June 4th, 1854, on leaving the chair as president of the society.)

GENTLEMEN OF THE SOCIETY,—

It is customary, on occasions like the present, to select as a topic for discourse some subject connected with the general interests of the profession, such as its history, its wants, its claims, or its duties. I hope to be pardoned for departing in some degree from this practice, and presenting before you a simple essay on a surgical subject. The reason for adopting this course is, a firm conviction that the first want, and greatest interest of the medical profession, is the improvement of medical science, and because I have made poisoned wounds, to which your attention is asked today, the subject of especial study, and entertain views concerning their treatment, which I believe to be both new and important.

The class of poisons to be considered is that, denominated by Orfila, septic, or putrid poisons. It embraces, among other substances, the venom of serpents, the woorara or American poison hastens death. This circumstance is not conclusive, as alcohol is of itself a poison for them.

3. Persons bitten by rattle-snakes when in a state of intoxication by alcohol are not, on that account, secure. I have authentic information of four cases in which the bite of that snake proved rapidly fatal on intoxicated persons. Another may be found related in the American Journal of the Medical Sciences, Vol, 8, 1831.

As not more than one in ten of the wounds made by the most venomous serpents proves fatal, a case of failure of a remedy is sufficient to counterbalance many cases of its success.

As the woorara is one of the poisons with which I have experimented, and the one which I propose to use to-day, it may be well, before going further, to say a few words on the subject of the nature of this substance. As usually met with, the kind which is brought from South America is contained in small gourds over the internal surface of which it is spread. On being detached it presents a dark color, has a resinous fracture, a bitter taste, is readily mixed with water, but imperfectly dissolved by it. Its appearance is the same when mixed with alcohol; but both these fluids dissolve the active principle of it. The solution is neither acid nor alkaline. If the quantity of water used be small, the mixture has a ropy, tenacious consistence. The solution is coagulated by the nitrate of silver, and by the solution of iodine and iodide of potash in distilled water, and, when treated with the latter solution, neither the part coagulated nor the fluid expressed from it retains its poisonous quality. It does not effervesce with acids. Its aqueous solution is not coagulated by heat, and boiling does not impair its activity.

The active principle has generally been considered as analagous to strychnine,* and Pereira states that the strychnos toxifera yields the basis of it.

Nearly all recent authors have copied the account of its manufacture given by Humboldt,† who enters into detail, and says

* Element of Materia Medica, Vol. II., p. 364.

† Voyage to the Equinoxial Region of of the New Continent, Vol. II., pp. 547 to 556.

it is made from the bark of the root of a species of *liane*, called by the natives of the banks of the Amazon, Bejuco de Mavacure.

"The chemical analysis of the woorara has been performed by Boussingault and Roulin, (Annales de Chimie, Sept. 1828,) who found in it a bitter principle, *very different from the strychnia* acetic acid, gum, red coloring matter, salts, etc.*

De la Condamine states that it is an extract made by heat from the juice of divers plants, about thirty in number, and that the *liane* is one of them.

Such was the state of our knowledge on this subject, when in 1850 Messrs. Pelouze and Bernard read a note at the Academy of Sciences on the subject.

Mr. Goudot, who furnished them with a specimen of the poison, confirms in general the account of Humboldt, as to the manner of its preparation from the juice of the *liane;* but he adds the important statement that "before the extract is quite dry they drop into it some drops of the venom of serpents collected from the vesicles of the most venomous species." Messrs. Pelouze and Bernard conclude that "the woorara acts upon animals in the manner of a venom."‡

From the discrepancy of these different accounts, it might be inferred that the various poisons prepared by the Indians of the Amazon and the Orinoco differ essentially in their properties; and Humboldt states that such is the case. There are, however, two facts stated by Humboldt himself which go to favor the opinion of Bernard and Pelouze. The first is that the juice of the *liane* before concentration is inocuous. The other is that "the Indians who have been wounded by poisoned arrows in war described to us the symptoms as entirely like those which are observed from the bite of serpents."

It is not probable that a juice from which a poison of such extraordinary activity is manufactured would be itself without power; and it is impossible to confound the effects of strychnia and those of the venom of serpents when applied to a wound on

* Dict. de Medicine, Tome IX., p. 483.

‡ Comptes Rendus de l'Academie des Sciences Seance Du, October 14, 1850.

the human subject. For myself, having made more than a hundred experiments with the woorara, I entertain but little doubt that the active principle of the specimens which I have made use of is the poison of serpents preserved in extractive and gummy matter. Those specimens were two. The first I believe to be a part of the same as that furnished to Messrs. Bernard and Pelouze by M. Goudot, as it was procured for Dr. J. W. Green and myself from M. Flourens at the Garden of Plants, by the Prince Charles Bonaparte. The other, which came from the Amazon, was furnished me by Dr. David Green, of New York. Their appearance and action were identical in every respect.

My reasons for believing its active principle to be the venom of serpents are these:

1. Its effects on birds and animals are strikingly like those preduced by the venom of the rattlesnake; and in many cases no difference can be perceived between them.

2. These effects are entirely unlike those produced by the vege table alkaloids.

3. Iodine neutralizes it as it does the venom of serpents, but has no such effect on vegetable alkaloids.

4. It is, like the venom of serpents, innocuous when taken into the stomach, except, perhaps, when used in very large quantities, or in circumstances very peculiar. This is not the case with any known vegetable poison.

5. It is well known that the poison used by the *North* American Indians for their arrows is that of the rattlesnake. I have learned this from such varied sources as not to leave any doubt on the subject. My inquiries have related to the Indians of California, New Mexico, and Texas, and been directed to medical officers of the army and intelligent travellers. The answers have never varied. The art of poisoning weapons is no longer a secret among them, but is often employed by those whites who adopt their customs. Dr. George Johnson, of St. Louis, who has traveled extensively on the Rio Grande, gave most accurate and valuable information concerning the habits of several Indian tribes in this respect. He states that there is a variety of rattlesnake on the Rio Grande, whose poison vesicle is much developed, and forms a prominent projection. This species is much sought for, on account of the quantity of virus contained in the sac.

I am, therefore, justified in stating that the poison used for arrows is, *in some instances*, the virus of serpents. There is great reason for believing that the process of preparing it is, as far as possible, kept a secret, except in what relates to the preparation of the juice of the plants destined to serve as a vehicle for the preservation of the venom.

If I am mistaken in this point, and the woorara should be found to contain some poisonous vegetable principle hitherto unknown, it will not invalidate my conclusion in regard to the effect of iodine upon it. It would only extend the application of this substance to the treatment of two kinds of poisoned wounds instead of restricting it to but one.

Having disposed of this point, I come now to the method of treatment which I propose for wounds poisoned by the woorara.

1st. It consists in the application of cups upon the part, or of ligatures around the member wounded, so as to arrest absorption.

2d. In injecting or infiltrating into the subcutaneous tissue the solution used as antidote.

The cups should be applied for a short time before the infiltration is employed, so as to fill the tissues with fluids, and prevent the injected liquid from producing abscess by mechanical injury. They should be allowed to remain on from five to ten minutes after the infiltration has been effected, in order to allow time for the antidote to come in contact with the poison before the latter has entered the circulation. In case much swelling and effusion have taken place before treatment can be applied, the application of cups would be unnecessary, as the fluid injected would in that case pass freely through the tissues without it. The strength of the solution should depend on the state of the parts, and the extent to which it is desired to disseminate it in the tissues. For a recent wound ten grains of iodine, and thrice that quantity of iodide of potassium to the ounce of distilled water should be employed. It should be put upon the wound, and the tissues filled with it for an inch around. When the swelling is already extensive, one-half or one-fourth the above strength will suffice; and the solution should, in that case, be disseminated as extensively as possible through the part affected, by introducing it, if necessary, at several different points. In a recent case I would advise one drachm of the solution to be injected.

In order to perform the injection or the infiltration perfectly, it is requisite to have cups to fit the inequalities of surface of the different parts of the trunk and members. Although ligatures can be made to arrest the circulation, and consequently the absorption, and fill the tissues with fluids, they do this less rapidly and less perfectly than cupping glasses. To perform the infiltration, small trochars like the exploring trochar are required, or they may be made still finer, (see plate 2, fig. 5 and 6). To the canula of the trochar a small syringe, like that called Anel's, should be adapted. (Plate 2, fig 4.) After the trochar has been introduced, and the stylet withdrawn, the syringe should be adapted to the canula; while the injection is gently pressed in, the cupping glass should be gradually exhausted. This is the method I advise on the human subject. When it is desired to employ it for experiment, a small syringe, the piston of which moves with a screw of which half a turn presses out a drop, and of which the point is also adapted to the canula, should be employed to insert the poison in the tissues. (See plate 2, fig. 7.)

I was led to the belief that iodine is the proper application to the snake bite, by experiment. Selecting salts which had not been used by others, I found the selection I have recommended, to be the only one which prevented the fatal effects of the poison, without producing an eschar. The solution of the iodide of potassium alone, has no effect as an antidote.

I am aware that the infiltration of the solution into the tissues is the part of the treatment which will be longest in receiving the assent of physicians, and the most difficult to introduce into general practice. Nevertheless, it is easy to understand, a priori, the reason and the necessity for so doing. In order to neutralize a poison once introduced into the system, it is essential that the antidote should be also introduced, and placed there where the poison exists, and in contact with it. Now, in case of an inoculated poison, any application which is confined to the external surface undertaken with the view of finding the antidote to the venom of the crotalus; and of testing the effect of such antidote, when thrown into the tissues of the animal body where the venom has been inoculated. They have furnished results, not before noticed by other observers, which are deemed of sufficient importance to be placed first in order.

The new facts observed are—

1. A spasmodic action of the whole muscular system, but more particularly of the larynx.

2. A certain change of the blood globules.

The antidote recommended is, the solution of iodine and iodide of potassium in water, applied upon the wound, and infiltrated into the tissues of the part affected.

As a means of preventing absorption, and rendering the action of the antidote more effectual, the use of cupping-glasses is recommended.

The serpent employed in these experiments was the massasagua, or prairie rattlesnake, the bite of which is deemed less dangerous than that of some other varieties. This probably does not arise from a difference in the venom, but from the snake being smaller and not sufficiently strong to make a deep wound. That such is the case, is proved by the fact that animals and persons bitten on the cutaneous surface, rarely die of the wound; but when bitten on the mucous surface, death is generally and speedily the result. In birds, also, whose skin is tender, the bite is uniformly and quickly fatal.

My experiments were mostly made on the domestic pigeon. The rapidity with which the poison affects it, and the size and form of its blood globules, render it peculiarly adapted for this purpose When freely bitten, the birds generally live from five minutes to two and a half hours, according to the extent of the wound and the degree of concentration of the poison. There is great difficulty in experiments with this venom, in arriving at the degree of accuracy required in all such cases. This difficulty arises from the impossibility of measuring accurately the quantity used, the strength of each specimen being liable to vary from that of others. Not only those procured from different species, but even specimens procured from the same species, and the same serpent at different periods, differ in strength. The season, the temperature, the food of the serpent, the frequency of discharge, and other causes difficult to appreciate, render the bite of any serpent most uncertain in its effects.

In order to obviate, as far as possible, the causes of uncertainty, a serpent was not allowed to bite more than three times the same day. One of the pigeons bitten was left without treatment, and if this did not die, all experiments with that serpent on that day were left out of consideration.

*Effects of the Poison.*—As an example of the effects of the poison, I transcribe from the record of experiments the following case, in which, from death occurring slowly, the changes could be carefully noted:

Friday, September 1, 1853.—A pigeon plucked under the wing was bitten at that point by a serpent. In fifteen minutes the wound and skin for half an inch around it were of a blue color. Effusion of serum had raised the skin, and separated it from the tissues beneath. At this time, slight spasmodic twitching of the fasciculi of the pectoral muscle beneath the bite was observed.

In twenty-two minutes this spasmodic action was greatly increased, and all the muscles, those of respiration especially, were affected with a tremulous movement, which passed off and recurred in paroxysms.

At this time the movements of the larynx in expanding and contracting were found to be much restrained. This organ being in full view in the pigeon when the mouth is opened, may be seen expanding during each inspiration, contracting after each expiration, and closing instantly and perfectly if it be touched, or if an effort be made to swallow.

In this case these movements were restrained, becoming more so as the effects of the poison increased. In thirty minutes there was difficulty of standing, the bird was gasping for breath, the mouth open, the eyes closed, the respiration spasmodic. At this time, the larynx was still more contracted, and the inspirations were at times accompanied by a sibilant sound.

In forty minutes, the bird was unable to stand, the respiration more irregular, the larynx more contracted. On being handled, this bird still moved its wings, but the legs were paralyzed.

It died at sixty-one minutes, the larynx being almost entirely closed for ten minutes before death. Observations on twelve birds which died, did not in any case show symptoms essentially varying from these, except in the greater rapidity or slowness of their occurrence. In some the spasm of the larynx was less, in others more decided. In one the larynx was found to dilate fully and spasmodically several times in succession, and then close perfectly so as apparently to cause the death of the bird.

The vision and the hearing were apparently unaffected until an advanced stage of the action of the venom.

The birds uniformly lose the use of the posterior extremities first, and one which recovered did not regain the power of using them for two days.

*Effects noticed in the parts bitten.*—Almost instantly, in rapid cases, the wound turns blue. This discoloration spreads rapidly, principally in the direction of the vessels leading to the heart. On laying open the wound, the tissues are found discolored, brown, infiltrated with serum, presenting all the appearances of a wound in approaching gangrene. If the wound be on an extremity gangrene actually occurs.

*State of the blood.*—When death is delayed, the blood found in the cavities of the heart and in the great vessels, is of a dark color, and not coagulated. When death occurs rapidly, the blood is found coagulated, but less firmly than in the natural state.

Felix Fontana states that the venom of the viper causes the blood to coagulate in the vessels and the cavities of the heart. Much as I admire the genius and patience of Fontana, who, notwithstanding his profession (he was an ecclesiastic), devoted himself to researches concerning a subject so repulsive as that of experimenting with vipers, and made over six thousand experiments with them, I think it is still permitted to doubt whether in this respect he is not mistaken. The viper is nearly allied to the crotalophorus, as this species of serpent is to the crotalus, and there is every reason to believe that in regard to the venom of these serpents, there exists only the difference which results from quantity and various degrees of activity. I should desire a new series of observations directed to the subject, before admitting that the venom of the viper produces effects on the blood directly the reverse of those resulting from the venom of the rattlesnake.

There is, probably, no disease or state of the system in which the fibrin of the blood is reduced to as small a quantity as it is in persons or animals bitten by these serpents.

As a consequence probably of this, ecchymosis occurs, and the member bitten, and sometimes the whole cutaneous surface, is covered with spots like those which are observed in petechiæ hemorrhagicæ. If death does not result in a few hours, hemorrhage often takes place. It may occur from the mucous surfaces, from ulcers, slight wounds, and from the bite itself. In the case of a patient admitted into the Illinois General Hospital on account of a bite, Dr. Johnson was unable to detect any appearances of fibrin in the blood which came from the mouth. In a dog bitten on the abdomen, a hemorrhage occurred, the next day from a slight punctured wound, which was near proving fatal.

*Microscopic examination of the blood.*—I have already stated that in extreme cases, the fibrin is entirely wanting, or at least is in so small quantity as not to be perceived by the microscope. The globules, the only other part of the blood subject to microscopic examinations, are in the pigeon more or less altered.

The venom having been extracted from a serpent and mixed with a few drops of distilled water, the mixture was added to the blood of a pigeon, under the focus of the microscope. As fast as the globules were reached by the venom they changed their form and assumed the appearance, shown in plate ii, fig. 3, of the accompanying paper.

A pigeon which had died from the bite of a rattlesnake, was examined fifteen hours after death. There was no appearance of putrefaction. Fig. 2, plate ii represents the appearance of the globules of blood taken from the vessels of the head.

I have examined them in numerous other instances, with the aid of my friends, Drs. H. A. Johnson and J. C. Morfit. In some of them we found, immediately after death, the blood of the great vessels much altered. It was not coagulated. Some of the globules were indented, others flattened on the side so as to be shield-shaped, others assumed a dumb bell form. In these cases many granules, resembling the nuclei of the globules, were noticed.

In the blood taken from the wound of a bird dead of the bite, Dr. Johnson found much granular matter and few globules. In that taken from the cellular tissue, at a certain distance from the bite, the globules were found thickened in their transverse, and shortened in their longest diameter. This latter change resembles that produced by a solution of sugar, and it did not result probably from the direct action of the poison, but from the globules having been macerated for some time in the effused serum.

When some hours had elapsed after the bite, before death, and the local effect was considerable, the blood taken from the vicinity generally showed an unusual number of white globules, which manifested a tendency to aggregation.

*Treatment of the bite.*—These is no point of practice in surgery which is less settled than the treatment of the bite of serpents. For reasons already alluded to, the wound inflicted even by the most poisonous species, such as the cobra de capello and the crotalus, are not commonly fatal. Hence, many inert remedies and others calculated to injure, have received the credit of curing, and come into popular use. Without, at the present time, going into this subject, it may suffice to state that the efficiency of no method has heretofore been established by direct experiment, if we except the application of cupping glasses. These, made use of as soon as the bite is inflicted, and continued for some time, have the effect of retarding the action of the poison. In cases where the dose is barely sufficient to kill, delaying its action neutralizes its effect, by causing it to be taken into the system in small doses.

*Tracheotomy.*—Bearing in mind the experiments of Dr. Marshal Hall, on dogs poisoned by strychnia, one of which was saved by tracheotomy, I no sooner observed that spasm of the larynx was one of the effects of the poison of the serpent than the operation presented itself as a possible means of relief. It was performed upon pigeons six times, in each case the effects were so far advanced as to show an undoubted tendency to a fatal termination. In no case did it prevent death, yet it must be admitted that in some of them it rendered the respiration regular and easy and evidently prolonged life.

*Treatment with the solution of iodine.*– In experimenting with different medicinal solutions, while searching an antidote to the effects of the bite of the rattlesnake, I was led to the belief that the solution of iodine and iodide of potassium in water, exercises a much greater influence over it than any other substance, excepting some of the most powerful caustics.

I extracted the virus from the vesicle of a serpent; it stood in a drop on the surface of a spoon. Having made superficial wounds on the breast of the two pigeons, I put the venom on them, dividing it between them, as nearly as possible, equally. On one of the wounds a few drops of solution of iodine was placed. It was of the strength of ten grains of iodine and thirty grains of iodide of potassium to the ounce of distilled water.

These birds were not closely watched, but at the end of six hours the one treated with the iodine was quite well, and the other was dead.

I repeated this experiment with much care, and having a larger quantity of poison used four pigeons. Of the two not treated, one lived two and the other three hours, those to which the solution of iodine was applied lived, and did not appear to be affected.

It is well known that wounds thus inoculated are much less rapidly fatal than the bite itself of the serpent. I therefore applied the solution upon the breast of a pigeon where a bite had been inflicted, but it did not prevent death from taking place in the usual time.

When we consider that the fangs of this serpent are capable of penetrating the tender skin and tissues of a pigeon at least half an inch, and are so constructed as to deposit the venom there, that the part of it which is left upon the surface is without effect. that an application to the surface can but slightly penetrate the wound, it becomes obvious that an antidote applied in that manner cannot reach the virus, and must of necessity remain without effect upon it. So far as the virus is concerned, it is the same as if the solution had remained in the bottle.

Under these circumstances, I thought of a method of treatment which I had made use of with success in some cases of erysipelas and oedema, viz: infiltrating the solution into the tissues. This operation is performed as follows:

A pigeon being prepared, and bitten as before directed, a cupping glass is immediately applied over the wound. A fine trochar being then passed beneath it and under the skin to the seat of the wound. the stylet is withdrawn and the solution of iodine injected through the canula into the tissues The cup is then continued upon the part for from five to ten minutes.

Of ten pigeons treated in this manner, there died five, recovered five. Average time of death, two hours and fifty-two minutes. Of ten birds bitten, without treatment, all died, average time of death, eighty-eight minutes. Of those which died after being treated with iodine, two had the wound so situated as not to be covered by the cupping-glass, and in all of them the solution used was too weak, being of but half the strength required and of that before directed to be used.

I also tried cupping alone, and the injection of a solution of the lact ferri, eight grains to the ounce of distilled water, and distilled water alone. Dr. Morfit, who was kind enough to assist me in my experiments, tried liquor potassa, a solution of bi carb. soda, tr. arnica, and a solution of ammonia in water.

The cupping alone has the uniform effect of retarding the action of the poison and prolonging life. In cases where the dose of poison is barely sufficient to kill without any treatment, this of itself is capable of preventing death. This was the result obtained by Magendie, and it is not a little surprising that a means of treatment of such certain efficiency should be so seldom resorted to in practice.

On the other hand, birds treated with the aqua ammonia died quickly. Fontana found that aqua ammonia, applied to a viper bite, hastened death. Yet this is the most popular application for serpent bite.

In regard to the other fluids tried, it is sufficient to say, in general, that none of them had an effect, except those capable of producing an eschar. Soda and potass do this, unless very weak. Fontana, who tried the mineral acids, and almost every imaginable solution in his experiments with the viper, found the caustic potash alone capable of preventing its effects.

The result of my experiments, taken in connection with those made by others, is, that up to the present time, no substance or solution has been found capable of preventing the fatal effects of the rattlesnake bite (unless destroying by caustic or excising the tissues of the part) excepting the solution of iodine. This, within certain limits, is capable of neutralizing it.

In order to guard against error, it should be stated that, in experimenting with birds, the separation of the skin by injecting even distilled water and applying cupping-glasses, is liable to cause the skin to fall off in scales. When the skin is not extensively separated, the solution recommended does not produce an eschar. The effects of iodine solutions on the tissues, since the publication of Velpeau's work on the subject, are so well understood that it is unnecessary to dwell further on this point.*

The effect of solutions of iodine, infiltrated into the tissues around the bite of the rattlesnake, is to prevent their discoloration and preserve the natural texture and color of the parts. Even when it does not prevent death from occurring, it still has this effect in a great degree.

The deductions which I think may legitimately be made from the foregoing facts, are—

1. The veuom of the crotalus produces spasm of all the muscles —most marked in the muscles of respiration.

2. This venom produces a peculiar change of the blood globules, which consists of alteration of form and disintegration.

3. If death is delayed, it deprives the blood of its fibrine.

4. The solution of iodine and iodide of potassium. in the proportion of ten grains of the former and thirty of the latter to the ounce of distilled water, is, within certain limits. an antidote to the venom of the rattlesnake.

5. When the venom is deeply inserted, or when it has been

* D's Injections Medicamenteuses dans les cavites closes. Par A. A Velpeau- Paris, 1846.

absorbed, the antidote, to be effectual, must be infiltrated into the tissues.

6. This infiltration can be performed without causing loss of substance, or producing either eschar or suppuration.

NOTE.—I am under great obligation to Drs. H. A. Johnson and J. C. Morfit, of Chicago, for aid in conducting experiments with serpents, making the microscopic observations and drawing the figures of the plates.

I am also deeply indebted to my young friend, Robert Kennicott, for procuring for me as many serpents as I needed for my experiments, and for many valuable facts in relation to the effects of the poison.

